# Physician-assisted Suicide in Serbia

**Published:** 2018-04

**Authors:** Božidar BANOVIĆ, Veljko TURANJANIN, Emir ĆOROVIĆ

**Affiliations:** 1. Dept. of Criminal Law, Faculty of Security Studies, University of Belgrade, Belgrade, Republic of Serbia; 2. Dept. of Criminal Law, Faculty of Law, University in Kragujevac, Kragujevac, Republic of Serbia; 3. Dept. of Criminal Law, Faculty of Law, State University of Novi Pazar, Novi Pazar, Republic of Serbia

**Keywords:** Physician-assisted suicide, Assisted suicide, Serbia, Legalization

## Abstract

**Background:**

Physician-assisted suicide is one of the features with very different legal solutions in the world. In Serbia, physician-assisted suicide is a crime, within a crime of the assisted suicide. The possibility of the legislation of the voluntary active euthanasia may open the door to the decriminalization of the physician-assisted suicide.

**Methods:**

Data were obtained from the Clinical Hospital Center in Kragujevac, Republic of Serbia collected during 2015. The research included 88 physicians: 57 male physicians (representing 64.77% of the sample) and 31 female physicians (35.23% of the sample). Due to the nature, subject, and hypothesis of the research, the authors used descriptive method and the method of the theoretical content analysis.

**Results:**

We have raised numerous questions. A slight majority of the physicians (58%) believes that physician-assisted suicide should be legalized, while 42% is for another solution. 90.9% took a viewpoint that it is completely unacceptable legalization of the physician-assisted suicide for all age groups, while the remaining 9.1% is to such legislative move. From the other side, 40.7% of respondents would prescribe a lethal dose of the medicament to the patient, who is in the terminal stage of the incurable disease, but, 59.3% would not do it. Interesting is that 13.8% of the physician had a patient who asked for the information how to commit suicide, and 12.5% gave them such information.

**Conclusion:**

Physicians in Serbia are divided on this issue. The majority of them are for the legalization of medical assistance to suicide, but there is a strong division among them on various issues.

## Introduction

Assisted suicide is one of the felonies whose criminalization varies from country to country. Suicide is no longer punishable by any comparative criminal law, but this tendency was the most opposed by the English legal system, where suicide is decriminalized in 1961 (1). In that time, the Government seized the person’s property, if he or she commits suicide, because they (2) deprived the king of one vassal in that manner. In the early American legislation, the attempted suicide was treated as a misdemeanor, but today, as well as in the rest of the world, it is treated as an unpunishable act. In addition, there is a possibility of psychiatric examination of the person who attempted suicide (3). However, inducing someone to suicide and assisting him with it is punishable in the majority of countries. In some US countries, it is equated with murder, while in others, like Michigan, it represents a privileged form of murder (3). In the comparative theory, there is a difference between assisted suicide and physician-assisted suicide in order to further qualify the aid in committing a suicide (4), whereby both procedures are related to the deprivation of life of the patient due to his serious health condition. In the first procedure, we have a perpetrator who is a third party and helps a patient to terminate life (assisted suicide), while in the second case, a physician (physician-assisted suicide) occurs as an assistant (5). It is common that a person is helped to commit suicide by doing something, but it is not uncommon to help him by not doing anything, which stands as one of the forms of passive euthanasia (6).

Living and dying in accordance with the person’s own beliefs and desires are considered to be one the greatest human freedoms, and one of the most common wishes of the patients who are in the terminal stage of the disease is to end their lives with a certain amount of dignity (7). This raised a question of one of the most important problems in the past and present times. It is an issue of decriminalization of euthanasia and physician-assisted suicide (8, 9). This also actualized the question of decriminalization of assisted suicide. The debate on these issues has not decreased for many years. Unlike euthanasia, in which a physician deprives the patient’s life by active engagement, at physician-assisted suicide (hereinafter: PAS), a doctor prescribes a medication that a patient will take when he decides to die. Therefore, PAS is an act by which a physician facilitates a patient’s death by providing to him necessary information and means to perform the act. PAS is somewhere in the middle between euthanasia and suicide and for some patients, it is only a way to avoid suffering and greater loss of control over their own body (10). As a primary argument in favor of PAS, we could find autonomy of the will and the right to the patient’s own will, then, PAS shows compassion and mercy, and ensures release from suffering (11, 12).

In Serbia, there are strong efforts for legalization of the euthanasia, and, in connection with it, PAS. Therefore, it was urgent to conduct research among physicians in Serbia on this topic. In this work, we will mainly deal with the PAS in Serbia, particularly in Kragujevac, where we conducted a study among physicians.

## Materials and Methods

The data were collected in Clinical Hospital Center in Kragujevac (Serbia), during the first half of the 2015 year. For the current analysis it has been derived from the broader research project whose aim was to identify occurrence, distribution, and opinions of the physicians about euthanasia and physician-assisted suicide. In this paper, we analyzed the main part of the obtained data. Research is primarily based on quantitative research approach, and data were collected using a short survey, created specifically for the purpose of this study.

In the civilized countries, today physicians are increasingly faced with demands to assist patients in committing suicide or to apply euthanasia (13, 14). In the connection with the efforts for euthanasia legislation, we conducted a survey among the physicians from Clinical Hospital Center in Kragujevac (Serbia). We analyzed the segments of the dataset which concern to ten questions, described in the following tables. To each question, we offered two answers: yes and no. Some of the questions are bound for each other.

All participants in this study expressed the informed consent to participate and they returned completed questionnaires in the closed envelopes. The university Ethics Committee approved the study.

The scope of the tested population, gender structure of the respondents, as well as the diversity of the health departments in which participants are employed, gives us the possibility of a wider generalization of the findings to the physicians’ populations across the whole country.

The initial sample plan was to try to conduct a survey of all employees in this medical institution. Of 100 physicians, 88 expressed their willingness to be participants. The final sample included 88 physicians: 57 male physicians (representing 64.77% of the sample) and 31 female physicians (35.23% of the sample). The study was divided into three parts: in the Ambulance, in the Emergency Room, while the third, which is at the same time the most numerous sample, included physicians from the departments of Surgery, Transfusion, and Cardiology. The initial hypothesis was that the physicians who work in the Emergency Room are prone to saving lives, and will be exclusively against PAS.

## Results

The sample included 88 physicians, who declared some PAS issues. The questions from the survey were:

***Question 1***: Could the suicide be an acceptable alternative for the patient who is in the terminal phase of the disease and suffers great pains?
***Question 2***: In the certain cases I would be willing to prescribe a dose of a medicament that will inevitably lead to the patient’s death, if a patient set a request in the terminal stage of the incurable disease and if such request is legal.
***Question 3***: Did you have in your carrier a patient (regardless of the fact is he in the terminal phase of the incurable disease), that ask from you information which medicament to use in order to commit suicide?
***Question 4***: Linked with the previous question, have you informed such patient how to commit suicide?
***Question 5***: You have a patient, who is 80 year old and have cancer. His pains are under control, but he thinks that he does not have any meaningful reason for life. So, he asks you to prescribe him enough quantity of pills that would lead to his death. Would you accept such request, if it is legal?
***Question 6***: You have a patient who has cancer, but his pains are unbearable. Would you accept his request for assisted suicide, if it is legal and he set such request?
***Question 7***: You have a patient who is mentally competent, suffers from the incurable disease in terminal phase and request from you in writing help in the suicide committing. Whether a physician has a right to administer a medicament to the patient that would lead to the patient’s death?
***Question 8***: Linked to previous question, would you prescribe such medicament to the patient, which he would use for suicide if it is legal?
***Question 9*** I support the law that would legalize PAS under fulfillment of the certain conditions.
***Question 10***: Do you support the legalization of the PAS for all age groups?
***Question 11***: Do you think that PAS should be legalized?

The answers are divided into three tables.

## Discussion

The PAS represent a very interesting issue through the worlds. In the midst of the world fight for the legalization of voluntary euthanasia, some American states decided to undertake the milder step, which is the decriminalization of assisted suicide, provided that it was carried out by a physician under the prescribed conditions.

Courts in some judgments (such *In re Conroy* and *In re Guardianship of Browning*) emphasize “highly sensitive nature of the right-to-die issue” (15, 16). Citizens across the United States began to support the right to a more dignified death wherein some data indicated that eight of ten Americans were convinced that a patient should have a choice to end his life under certain circumstances, and 55% of them were convinced that the moral right of the patients is to commit suicide (17, 18). Assisted suicide in Germany is not regarded as a criminal act. In this way, Germany has centered its position between the countries in which euthanasia and physician-assisted suicide are legalized and the others (8) where these two procedures are felonies (9, 19). Opening of the branch of the Swiss organization *Dignitas* (20) has increased a discussion about physician-assisted suicide (19). In recent years, more than 80% of the population supports euthanasia (21). In Germany, courts believe that there is no felony, even in cases of active euthanasia (22).

Assisted suicide in Serbia is considered a criminal offense by the article 199 of the Criminal Code, entitled Inducement to suicide and assisted suicide. Thus, the same article regulates assisting and inducing the person to commit a suicide. Among provisions that regulate offenses against public health there is no a separate criminal act of PAS, as in some other legislation, which redirects us to the crime of assisted suicide. Therefore, the perpetrator of this criminal act may be any person and it is irrelevant whether it is a physician, who is subject to the criminal liability as well as any other person. The basic form of the felony is encouraging or aiding someone to commit suicide, and the act itself is attempted or committed. The easier form of the assisted suicide is related to the assisting in suicide to the person which fulfills conditions for the euthanasia. If someone assists in suicide to the juvenile, or to a person who is in a state of the considerably diminished mental capacity, he will commit a more severe form of this felony punishable by imprisonment from two to ten years, but if someone assists in suicide to a child or mentally incompetent person, it represents the most severe form, punishable by imprisonment from at least ten years or 30–40 yr. Therefore, intention is necessary for the criminal responsibility of the offender is, whereby awareness of the perpetrator has to encompass the fact that assisting is done against the minor or a person who is in a state of the considerably diminished mental capacity or mentally incompetent person (23). Finally, there is a special form of the offence in cases of the cruel and inhuman treatment of the person who is in any kind of subordination or dependence to the defendant (24).

Starting question in our survey showed a sharp division between physicians about suicide. Only 51.1% consider PAS as an acceptable alternative for the patient who is in the terminal phase of the disease, but 48, 9% have an opposite attitude (Table 1). Physicians in ambulance are strongly divided, so we have 10 physicians with answer *YES* and 9 with answer *NO*; in the Emergency Room almost every physician is against suicide (89.47%), while in the third department 66% of the physicians are for the suicide in such cases, but 34% is not for that solution. The majority of the respondents would not be willing to prescribe a medicament that would lead to the patient’s death, even in the case that such procedure is legal and the patient is in the terminal phase of the incurable disease, which is visible from question 2 of Table 1. In this case, just in the departments of the Cardiology, Surgery, and Transfusion, a small majority of the physicians (56%) would be willing to prescribe a medicament, while the others would not do it. In the Ambulance, 33.3% is for prescribing, but 66.4% is not, while in the Emergency Room just one respondent has been for the positive answer. The next two questions are mutually linked. Therefore, on the third question, we have received answers that 13.8% of the respondents had a patient who asked him information which medicament to use to commit suicide. The majority of the cases happened in the most numerous department of our study (11.36%), but just 2.27% in the Emergency Room and 0% in the Ambulance, which is not surprising for the Ambulance, because patients retain for a short period of time. However, 12.5% of the physicians informed a patient, which medicament to use. Such act, according to the Serbian Criminal Code, is a crime. Especially interesting is the fact that from the questionnaires are visible that some of the physicians informed a patient about a medicament in a case where a patient did not ask for such kind of the information.

**Table 1:** Distribution of answers for the Questions 1–4

			***Frequency***	***Percent***	***Valid Percent***	***Cumulative Percent***
Question 1	Valid	YES	45	51.1	51.1	51.1
		NO	43	48.9	48.9	100.0
		Total	88	100.0	100.0	
Question 2	Valid	YES	35	39.8	40.7	40.7
		NO	51	58.0	59.3	100.0
		Total	86	97.7	100.0	
	Missing	System	2	2.3		
	Total		88	100.0		
Question 3	Valid	YES	12	13.6	13.8	13.8
		NO	75	85.2	86.2	100.0
		Total	87	98.9	100.0	
	Missing	System	1	1.1		
	Total		88	100.0		
Question 4	Valid	YES	11	12.5	12.5	12.5
		NO	77	87.5	87.5	100.0
		Total	88	100.0	100.0	

Table 2 consists answers to the Questions 5–8. In the Questions 5 and 6, we set similar situations. In the first one, the patient has cancer with pains under control. In that case, 36.4% respondents would accept the patient’s request for the assist in dying, and a majority in every department is to such conduct of the physician (72% against 28% in Ambulance, 0% against 100% in the Emergency Room and 48% against 52% in the third department). The situation is quite different in the next situation, where the patient suffers unbearable pains. In that case, a slight majority of the respondents would not accept patient’s request for PAS (50.6%), in contrast to the 49.4% that would. Even in the Emergency Room we have increased number of the physicians that are for the PAS (15.78% against 84.22%), while in the other departments physicians are for the PAS (in the Ambulance 52.63% against 47.37% and in the most numerous department 61.22% against 38.78% (we have to note that one respondent did not answer to the question). When we look at the answers to the Question 7, physicians believe that they should be someone who will take a patient’s life, not patients alone. In the first situation, 44.3% of the respondents believe that physician has a right to administer a lethal dose of the medicament to the patients, while in the other situation just 33% has an opinion that they would prescribe such medicament to the patient. In the first case 42.1% physicians in the Ambulance would do it, 60% in the department of the Surgery, Transfusion, and Cardiology, and 5.26% of the Emergency Room, in contrast to the 31.5% in the Ambulance, 0% in the Emergency Room and 46% in the other departments in the second case. Therefore, we may conclude that physicians believe that taking the patient’s life should be medical procedure that cannot be left to the patient, where doctors would just give a prescription for the lethal dose of the medicament.

**Table 2:** Distribution of answers for the Questions 5–8

			***Frequency***	***Percent***	***Valid Percent***	***Cumulative Percent***
**Question 5**	Valid	YES	32	36.4	36.4	36.4
		NO	56	63.6	63.6	100.0
		Total	88	100.0	100.0	
**Question 6**	Valid	YES	43	48.9	49.4	49.4
		NO	44	50.0	50.6	100.0
		Total	87	98.9	100.0	
	Missing	System	1	1.1		
		Total	88	100.0			
**Question 7**	Valid	YES	39	44.3	44.3	44.3
		NO	49	55.7	55.7	100.0
		Total	88	100.0	100.0	
**Question 8**	Valid	YES	29	33.0	33.0	33.0
		NO	59	67.0	67.0	100.0
		Total	88	100.0	100.0	

The answers to the last three questions, related to the legal themes, are visible in Table 3. In the beginning, we asked physician are they for the legalization PAS under fulfillment certain conditions. Answers showed a great divide among respondents. A small majority was against PAS legalization (52.3%), while 47.7% were for legalization. As it is a case with the other issues, on the final results a big influence has Emergency Room, where we have just 2 physicians who for legalization, while in the other departments, the majority is for such law. Further, the critical question was related to the PAS for all age groups. As we know, Belgium allowed euthanasia for all age groups (25), so we wanted to know the attitudes of the physicians in Serbia on the issue of the PAS. Our primary hypothesis was confirmed: just 9.1% of the respondents had been for the positive answer, while 90.9% were against such solution (in this case, 100% of the respondents in the Emergency Room and Ambulance were against such law). The eleventh question in this study was: Do you think that PAS should be legalized? Although their opinions were divided, the majority of physicians pleaded for the legalization of this procedure. From the following question 58% of physicians are for the legalization, and 42% are for the opposite solution. The results of the survey by departments have come as a sort of surprise. Namely, we expected that physicians who work in the Ambulance and in the Emergency Room would be absolutely against any form of deprivation of life, including PAS. The results in other departments, however, in which the physicians were mostly for PAS despite their divided opinions, were expected. In the first-mentioned two departments, although respondents’ opinions were divided, the number of PAS supporters prevails by only one respondent. We expected that the number of supporters in these departments would be significantly smaller since these are physicians who stand at the front line in the struggle for the saving patients’ lives, and that they will be more prone to the procedures that are not suicidal. Here, in both departments, we have the same percentage: 52.3% is for legalization, while 47.7% has a diametrically opposite attitude. However, this is not a large sample; these data are relative to a certain extent.

**Table 3:** Distribution of answers for the Questions 9–11

			***Frequency***	***Percent***	***Valid Percent***	***Cumulative Percent***
**Question 9**	Valid	YES	42	47.7	47.7	47.7
		NO	46	52.3	52.3	100.0
		Total	88	100.0	100.0	
**Question 10**	Valid	YES	8	9.1	9.1	9.1
		NO	80	90.9	90.9	100.0
		Total	88	100.0	100.0	
**Question 11**	Valid	YES	51	58.0	58.0	58.0
		NO	37	42.0	42.0	100.0
		Total	88	100.0	100.0	

## Conclusion

Assisted suicide has lately become an increasingly frequent topic in discussions, with the development of PAS in some comparative legislation systems. In Serbia, the assisted suicide is a crime, and there is still no distinction according to the fact if the perpetrator is a doctor or any other individual. The survey we conducted among physicians in Kragujevac has shown that the majority of them are for the legalization of physician assistants to suicide, but there is a strong division among them on various issues. In this research, we did not consider the ethical side of the PAS (26). The basic principle, on which this procedure is based, is to allow the patient the right to his or her own decision, at the same time protecting all his rights as a patient as well as protecting them from potential abuse.

## Ethical considerations

Ethical issues (Including plagiarism, Informed Consent, misconduct, data fabrication and/or falsification, double publication and/or submission, redundancy, etc) have been completely observed by the authors.
